# Glucose-dependent regulation of pregnane X receptor is modulated by AMP-activated protein kinase

**DOI:** 10.1038/srep46751

**Published:** 2017-04-24

**Authors:** Peter O. Oladimeji, Wenwei Lin, C. Trent Brewer, Taosheng Chen

**Affiliations:** 1 Department of Chemical Biology and Therapeutics, St. Jude Children’s Research Hospital, Memphis, Tennessee 38105, USA; 2 Integrated Biomedical Sciences Program, University of Tennessee Health Science Center, Memphis, Tennessee, USA

## Abstract

Pregnane X receptor (PXR) is a xenobiotic receptor that regulates the detoxification and clearance of drugs and foreign compounds from the liver. There has been mounting evidence of crosstalk between the drug metabolism pathway and the energy metabolism pathway, but little is known about this cross-regulation. To further delineate the energy metabolism and drug metabolism crosstalk in this study, we exposed HepG2 cells to varying glucose concentrations. We observed that PXR activity was induced under high-glucose conditions. This finding is consistent with previous clinical reports of increased drug clearance in patients with untreated diabetes. We demonstrated that AMP-activated protein kinase (AMPK) modulates PXR transcriptional activity and that pharmacologically manipulated AMPK activation exhibits an inverse relation to PXR activity. Activation of AMPK was shown to downregulate PXR activity and, consistent with that, potentiate the response of cells to the drug. Taken together, our results delineate a hitherto unreported axis of regulation that involves the energy status of the cell, PXR regulation, and drug sensitivity.

The sensitivity of patients to drugs depends on the pharmacokinetics of the drugs and particularly on the bioavailability of the drugs in the body. Orally administered drugs must pass through the intestinal wall and the portal circulation to reach the liver, and both the intestine and the liver are sites of first-pass metabolism^1^. Thus, many drugs may be metabolized before they attain optimal plasma concentrations or before they reach the target tissues, resulting in diminished efficacy. Drug metabolism is regulated by numerous drug-metabolizing enzymes (DMEs) and drug transporters such as cytochrome P450 (CYP) enzyme 3 A, CYP2B, CYP2C, aldehyde dehydrogenase, alcohol dehydrogenase, carboxylesterase, UDP-glucuronosyltransferase (UGT) 1A1, sulfotransferase, multidrug resistance protein (MDR) 1, ATP-binding cassette transporter C (ABCC) 2, and organic anion transporting polypeptide (OATP) 2 (refs 2, 3, 4, 5, 6). Differential expression of these DMEs and drug transporters is, therefore, a major determinant of the variability in drug response between individuals^7^.

The nuclear receptor pregnane X receptor (PXR; NR1I2), which is predominantly expressed in the liver and intestine, is regarded as a master xenobiotic-regulated transcription factor that modulates the induction of many genes, such as the aforementioned hepatic enzymes and transporters, that are involved in the hepatic drug-clearance system. Hence, PXR mediates the detoxification and elimination of drugs and other xenobiotics^8^.

Foods that contain complex mixtures of phytochemicals, such as fruits, vegetables, herbs, spices, and teas, have great potential to induce or inhibit the activity of DMEs, although dietary macroconstituents such as protein, fat, and carbohydrate ratios and total energy intake can also regulate DMEs^9^. The most abundant hepatic and intestinal phase I enzyme is CYP3A4, which metabolizes approximately 50% of marketed drugs. It is also a primary transcriptional target of PXR^9^; therefore, its activity is modulated by a variety of factors, including food components.

There is increasing evidence that drug metabolism can be affected by energy metabolism and *vice versa*. PXR has been implicated as having a complex role in energy homeostasis, as evidenced by its involvement in both lipid and glucose metabolic pathways^10^,^11^,^12^. In type I diabetes, a major manifestation of the metabolic syndrome, drug metabolism is affected by significantly increased drug clearance in untreated patients. but this effect can be reversed by insulin treatment^13^,^14^. This observation is consistent with studies conducted in a mouse model of type I diabetes, in which the mice exhibited increased CYP2B10 basal expression that was corrected by insulin administration^15^. These data suggest that changes in glucose homeostasis have clinically relevant consequences with regard to the hepatic metabolism of drugs. The clearance of any compound in humans is markedly dependent on the relative expression and activity of the various DMEs and transporters^16^; therefore, it is imperative to consider the potential modulation of PXR by food components (especially energy sources) and drugs, as such modulation may result in unfavorable long-lasting drug-drug interactions and probably fatal toxicity or in insensitivity to drugs, depending on many factors associated with the enzymes, drugs, and patients.

The heterotrimeric AMP-activated protein kinase (AMPK) is a monitor of cellular energy status in most eukaryotic cells and is activated under stress, such as that resulting from starvation, exercise, or hypoxia, which depletes the cellular ATP supply and thereby increases the AMP:ATP and ADP:ATP ratios^17^,^18^. The binding of AMP to AMPK causes a subsequent phosphorylation of the AMPK catalytic α-subunit at Thr172 by the upstream liver kinase B1 (LKB1), which leads to AMPK activation. Activated AMPK then regulates its downstream targets in order to inactivate ATP-consuming anabolic processes and activate ATP-generating catabolic processes^19^,^20^,^21^. In essence, the main function of AMPK is to detect energetic stress and restore energy homeostasis in cells. AMPK is regarded as a tumor suppressor in various cancers because of its inhibitory effects on anabolic processes such as protein and lipid synthesis, cell cycle progression, and cell growth, all of which are important for tumor development^22^,^23^. It is also important to note that upstream kinases of AMPK, namely LKB1 and calcium/calmodulin-dependent kinase kinase (CaMKK), are important tumor suppressors and are often mutated or dysregulated in various cancers^24^,^25^,^26^,^27^. Together, these factors make AMPK a potential target for cancer therapy.

Despite our knowledge of the cross-regulation between drug metabolism and energy metabolism, the molecular pathways of this interaction remain vague. The gluconeogenic transcription factor forkhead box transcription factor O1 (FoxO1) regulates the transactivation of both constitutive androstane receptor (CAR) and PXR target genes^28^. Also, AMPK regulates CAR, but the exact nature of this regulation remains unclear^29^,^30^,^31^. To further delineate the crosstalk between the drug metabolism and energy metabolism pathways, we examined the glucose-dependent regulation of PXR in this study. We identified AMPK as an energy status–dependent negative modulator of PXR transcriptional activity and associated xenobiotic metabolizing activity, even though PXR was found not to be a direct target of AMPK. We show that AMPK activation sensitizes liver cancer cells to drugs by downregulating PXR activity.

## Results

### Glucose regulates PXR transcriptional activity

**Figure 1:**
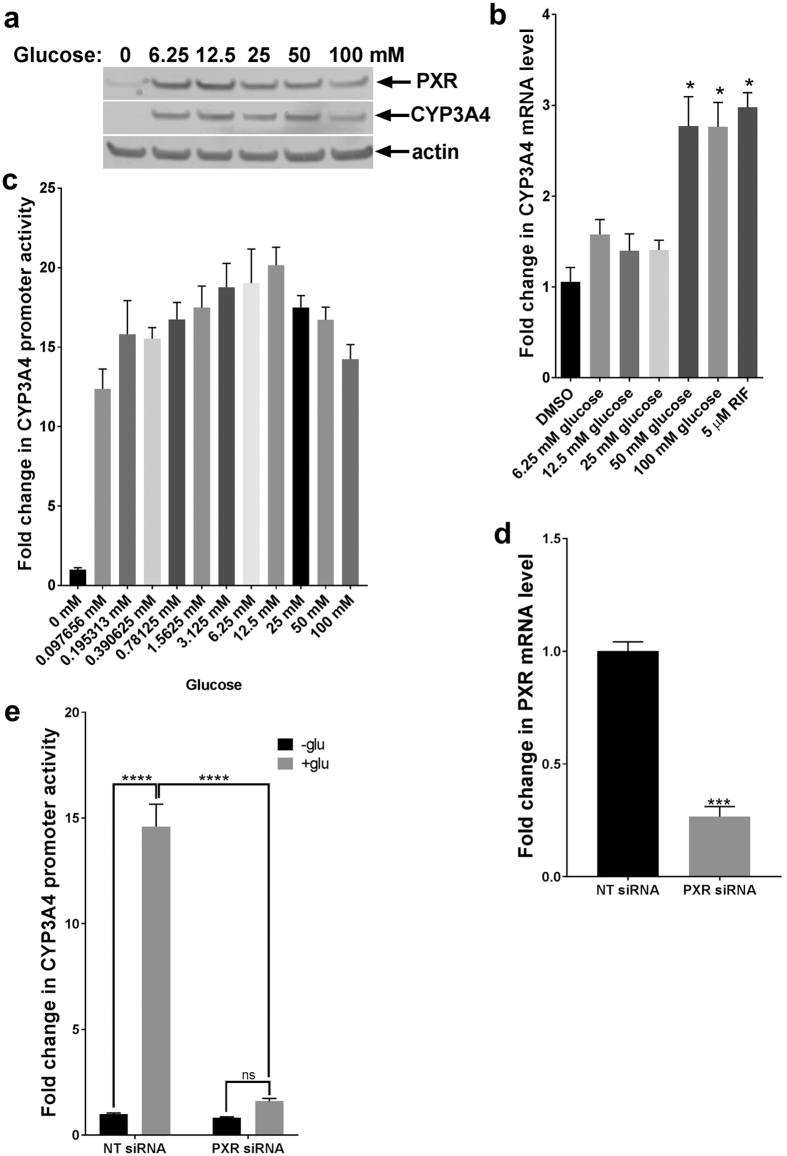
Glucose regulates CYP3A4 induction through PXR. HepG2 cells were deprived of serum and glucose for 24 h. Glucose was then re-introduced at increasing concentrations for 24 h, after which the PXR and CYP3A4 protein levels (**a**) and CYP3A4 mRNA levels (**b**) were assessed. Glucose stimulated CYP3A4 induction. (**c**) HepG2 PXR CYP-luc cells were treated as previously described. Luciferase activities were measured 24 h after treatment with increasing concentrations of glucose. (**d**) HepG2 cells stably expressing hPXR and CYP3A4-luciferase reporter (PXR clone 1) were transfected with either nontargeting siRNA (NT siRNA) or siRNA targeting PXR (PXR siRNA). The graphs represent the PXR knockdown efficiency (**e**). HepG2 PXR CYP-luc cells were transfected with either nontargeting siRNA (NT siRNA) or PXR siRNA. The cells were deprived of glucose and serum for 24 h then treated with or without 50 mM glucose, and the luciferase activity was measured 24 h after treatment. Luciferase assays were performed using Dual-Glo luciferase reagent, and the luciferase activities are represented as the fold change compared to untreated control cells. The values represent the means of three independent experiments, and the bars denote the standard error of the mean (SE). The *P*-values were determined using ANOVA with Tukey’s HSD test. *P < 0.05, ***P < 0.001, ****P < 0.0001 ns, not significant.

The previously reported evidence that drug clearance was significantly increased in patients with untreated type I diabetes, an effect that was reversed by insulin treatment^13^,^14^, coupled with evidence from the mouse model of diabetes in which mice exhibited increased basal expression of CYP enzyme that was corrected by insulin administration^15^, prompted us to assess the direct regulation of PXR by glucose *in vitro*. For this purpose, we starved hepatocellular carcinoma HepG2 C3A (HepG2) cells of glucose and serum for 24 h in glucose-free DMEM then treated them with varying concentrations of glucose for an additional 24 h. Reintroducing glucose to the cells increased the mRNA and protein levels of CYP3A4, a PXR transcriptional target (Fig. 1a and b). While 6.25 mM glucose was sufficient to increase CYP3A4 protein level (Fig. 1a), an increase in CYP3A4 mRNA level was not observed until the glucose concentration was increased to 50 mM glucose (Fig. 1b). It is possible that relatively low glucose concentration is sufficient to stabilize existing protein but for transcriptional upregulation, a high glucose concentration is required; we have therefore used 50 mM of glucose for most of the experiments in this study. We then assessed the promoter activity of *CYP3A4* in HepG2 PXR CYP-luc cells (described in the Methods section) and showed that the promoter activity was increased by glucose treatment in a dose-dependent manner and peaked at 12.5 mM glucose (Fig. 1c). To exclude the possibility that this phenomenon was cell-line specific, we conducted similar experiments in parental HepG2 cells, the colorectal adenocarcinoma cell lines LS180 and Caco2, and human primary hepatocytes. We observed that glucose upregulated CYP3A4 mRNA in each case; however, the effect in human primary hepatocytes was not significant and was very modest compared to that in the cancer cell lines tested (see Supplementary Fig. S1). To determine if PXR was involved in the glucose-dependent regulation of CYP3A4, HepG2 PXR CYP-luc cells were transfected with either control siRNA (NT siRNA) or siRNA targeting PXR (PXR siRNA) for 48 h to knock down PXR (Fig. 1d). After glucose and serum starvation, treatment of control cells with 50 mM glucose activated the *CYP3A4* promoter, which was strongly inhibited with PXR knockdown (Fig. 1e). This suggests that the effect of glucose on CYP3A4 induction is mediated by PXR.

To exclude the possibility that the effects of high glucose is nonspecific, we assessed the levels of CAR and other transcription factors in response to high glucose. We found that CAR is also upregulated to a similar extent as PXR; however, the mRNA levels of the other transcription factors assessed did not change with increased glucose concentration (see Supplementary Fig. S2). This suggests that CAR may participate in the downstream effects of high-glucose.

### AMPK negatively regulates PXR activity

**Figure 2:**
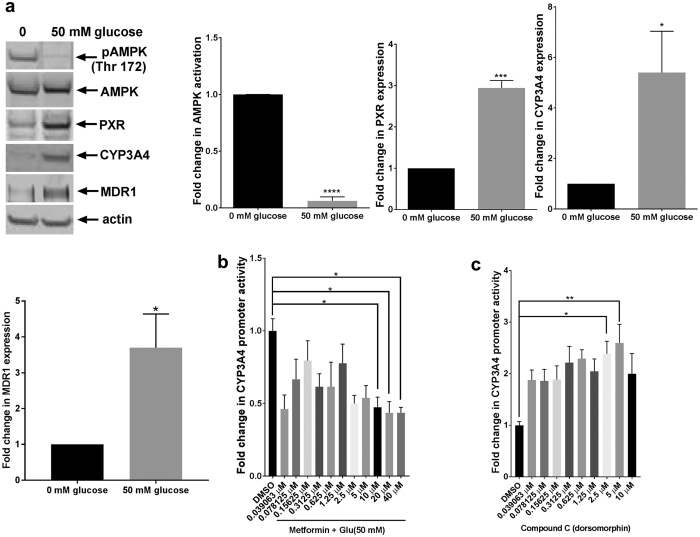
AMPK activation is inversely correlated with PXR activation. (**a**) HepG2 cells were deprived of serum and glucose for 24 h. The cells were then treated with or without 50 mM glucose. Whole-cell lysates were probed with antibodies as indicated (left panel). AMPK activity (pAMPK Thr 172) was normalized to total AMPK expression. Other protein expression quantifications were normalized to actin. HepG2 PXR CYP-luc cells were deprived as described in the legend for Fig. 1, and luciferase activities were measured 24 h after the cells were treated with glucose and metformin (an AMPK activator) (**b**) or compound C (an AMPK inhibitor) (**c**). The *P*-values were determined using ANOVA with Tukey’s HSD test: *P < 0.05, **P < 0.01, ***P < 0.001, ****P < 0.0001, n = 3. Results are expressed as the mean ± SE.

AMPK is the energy sensor in cells that functions to restore energy balance during periods of ATP depletion^21^. In an attempt to understand the link between glucose and PXR regulation, we examined the activation status of AMPK in HepG2 C3A cells under starvation conditions and in the presence of 50 mM glucose. AMPK was activated, as detected by the phosphorylation of threonine 172, when cells were glucose starved but was inactive in the presence of glucose (Fig. 2a). This is consistent with the results of a previous study showing rapid activation of AMPK and PKA in glucose-deprived hepatocarcinoma cells^32^. Interestingly, we observed an inverse correlation of PXR activity: PXR activity was downregulated under glucose-starvation conditions but upregulated in the presence of glucose, as determined by the increase in the protein levels of CYP3A4 and MDR1 (both of which are PXR transcriptional targets) (Fig. 2a). This inverse correlation led us to postulate that AMPK negatively regulated PXR. To test this hypothesis, we treated HepG2 PXR CYP-luc cells with 50 mM glucose and increasing concentrations of metformin—previously reported to be an AMPK activator^33^,^34^. We observed that PXR activity was impeded by AMPK activation in a dose-dependent manner (Fig. 2b). To confirm this observation, HepG2 PXR CYP-luc cells were starved of glucose to minimize all PXR activity and then treated with increasing concentrations of dorsomorphin—a known AMPK inhibitor^35^,^36^. PXR activity increased in a dose-dependent manner in response to increasing concentrations of dorsomorphin (Fig. 2c). Taken together, these observations suggest that the activation of AMPK represses the activity of PXR.

### Identification of AMPK modulators

**Figure 3:**
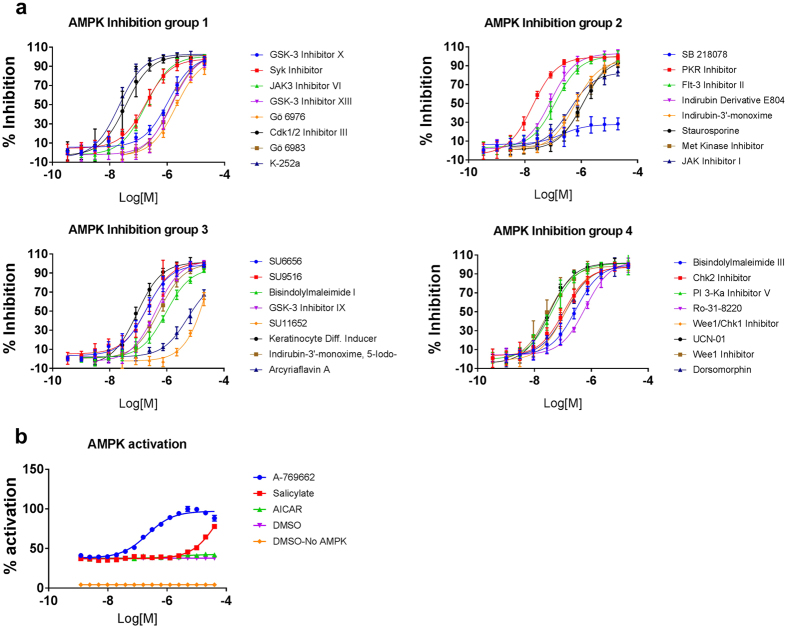
Screening of potent AMPK activators and inhibitors. The Calbiochem Kinase Inhibitor Library I and Calbiochem Kinase Inhibitor Library III were screened for potent AMPK inhibitors by using an *in vitro* AMPK kinase assay coupled with an ADP-Glo assay. (**a**) A total of 244 compounds were screened, and 32 compounds displayed more than 80% inhibition at a concentration of 2 μM, as compared to DMSO. (**b**) A-769662 and salicylate were identified as potent AMPK activators in the ADP-Glo assay.

Dorsomorphin (6-[4-(2-piperidin-1-ylethoxy)phenyl]-3-pyridin-4-ylpyrazolo[1,5-a]pyrimidine) is the only agent reported to be used as a cell-permeable AMPK inhibitor. Unfortunately, dorsomorphin (also known as compound C) is not specific for AMPK; it also inhibits the BMP pathway^37^,^38^ and a number of kinases other than AMPK^39^,^40^. The lack of a specific AMPK inhibitor prompted us to identify additional and more potent, cell-permeable AMPK inhibitors. To this end, we performed our primary compound screen with a library of 244 chemicals previously identified as kinase inhibitors. In an *in vitro* kinase assay coupled with an ADP-Glo assay (the latter assay measures the generation of ADP from the kinase reaction), we treated recombinant active AMPK with varying concentrations of the test compounds in 384-well plates and measured the ADP-mediated luminescence. Of the compounds tested, 32 that displayed more than 80% inhibition of AMPK at a concentration of 2 μM, as compared to DMSO without AMPK protein (Fig. 3a), were selected for analysis to determine their IC_50_ values (Table 1). We selected three compounds, the indirubin derivative E804 (ID E804), Cdk1/2 inhibitor III, and UCN-01, for further validation based on their potent inhibition of AMPK (Fig. 3a and Table 1) and cell permeability^41^,^42^,^43^. The ability of these three compounds to inhibit AMPK was assessed in a cell-based assay, and only ID E804 and Cdk1/2 inhibitor III inhibited AMPK activity effectively (see Supplementary Fig. S3). We also further characterized reported AMPK activators. A769662 and salicylate, both are known AMPK activators^44^,^45^,^46^, displayed dose-responsive activation of AMPK in the biochemical ADP-Glo kinase assay. AICAR (5-aminoimidazole-4-carboxamide ribonucleotide), another well-known cellular AMPK activator^47^, was included in our assay but did not activate AMPK *in vitro* (Fig. 3b). A similar observation was previously made by Hawley *et al*., who found that metformin did not activate AMPK in a cell-free system^34^. These results suggest that A769662 and salicylate are allosteric activators of AMPK and that AICAR may activate AMPK indirectly by decreasing cellular ATP and increasing AMP. All three activators were assessed for AMPK activation in a cell-based assay, and all three effectively increased AMPK activity (see Supplementary Fig. S3).

### AMPK kinase activity is essential in the regulation of PXR

**Figure 4:**
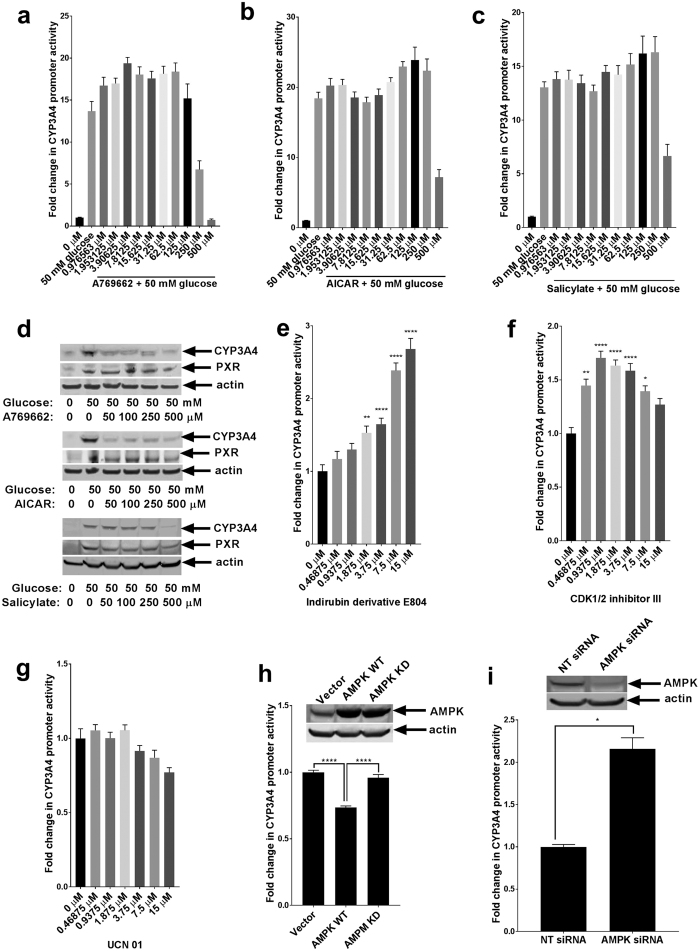
AMPK modulates PXR activity. HepG2 PXR CYP-luc cells were deprived as described in the legend for Fig. 1. Cells were co-treated with increasing concentrations of AMPK activators and 50 mM glucose. *CYP3A4* promoter activity was assessed by luciferase assay (**a–c**), or PXR and CYP3A4 protein expression were assessed (**d**). (**e–g**) Deprived HepG2 PXR CYP-luc cells were treated with increasing concentrations of indicated AMPK inhibitors, and luciferase assays were performed. Wild-type AMPK (AMPK WT), kinase-dead AMPK (AMPK KD), or control vector (Vector) were expressed in HepG2 PXR CYP-luc cells (**h**), or AMPK was knocked down in cells using siRNA targeting AMPK (AMPK siRNA) or NT siRNA as control (**i**), and cells were deprived for 24 h. Luciferase activities were measured 24 h after treatment. The *P*-values were determined using ANOVA with Tukey’s HSD test: *P < 0.05, **P < 0.01, ***P < 0.001, ****P < 0.0001, n = 3. Results are expressed as the mean ± SE.

To confirm the inhibitory effects of AMPK on PXR (Fig. 2), we used the AMPK activators A769662, AICAR, and salicylate. We starved HepG2 PXR CYP-luc cells of glucose then treated them with 50 mM glucose and increasing concentrations of the test compounds. Similar to our previous observation, AMPK activation inhibited PXR activity as assessed by *CYP3A4* promoter inhibition (Fig. 4a–c). AMPK activation also inhibited glucose-induced CYP3A4 protein expression (Fig. 4d). To confirm that the observed effects of the activators were not the result of cytotoxicity, we performed a cell viability assay and observed that at the concentrations tested, the compounds had no significant effect on cell viability (see Supplementary Fig. S4a–c). Conversely, the inhibition of AMPK by ID E804 and Cdk 1/2 inhibitor III activated PXR (Fig. 4e–f). PXR binds many structurally diverse compounds^48^. To determine if the effects of the AMPK inhibitors on PXR activation were the result of direct binding and activation of PXR, we tested the direct binding of the AMPK inhibitors to PXR by using a time-resolved fluorescence resonance energy transfer (TR-FRET) assay^49^,^50^. As shown in Supplementary Fig. S4d, all three inhibitors tested bound, but more weakly than the known potent PXR ligand T0901317^51^. It is important to note that although UCN-01 weakly bound PXR in our binding assay, UNC-01 did not induce PXR activity (Fig. 4g), which is consistent with its failure to activate AMPK in a cell-based assay (see Supplementary Fig. S3). This observation suggests that the activation of PXR by ID E804 and Cdk 1/2 inhibitor III was not mainly due to their binding to PXR where they may act as agonists, rather the activation of PXR observed is through the modulation of AMPK activity. Overexpression of WT AMPK, but not of the kinase-dead mutant of AMPK (AMPK KD), recapitulated the effects of the AMPK activators by causing a decrease in *CYP3A4* promoter activity (Fig. 4h). These results suggest that the kinase activity of AMPK is essential for its regulation of PXR activity. An siRNA-mediated knockdown of AMPK increased PXR activity, supporting our observations with AMPK inhibitors (Fig. 4i).

### PXR is not a direct target of AMPK

**Figure 5:**
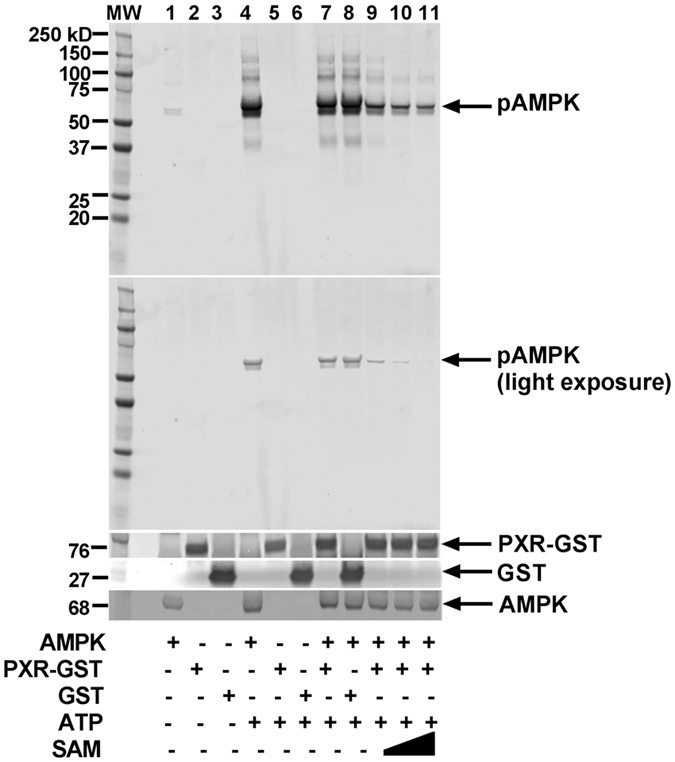
PXR is not a direct target of AMPK. Active AMPK was subjected to *in vitro* kinase assay with PXR and SAM peptide as substrates in the presence of nonradioactive ATP. The incorporation of phosphate into the substrate was determined by Western blot analysis with anti-phosphorylated serine/threonine antibody (pSer/Thr) (upper two panels). The lower three panels show the same blot reprobed with the indicated antibodies to detect PXR-GST, GST, or total AMPK.

AMPK activation has previously been shown to promote phosphorylation and regulate the localization of constitutive androstane receptor (CAR) in cells^29^,^30^,^31^. Therefore, we performed an *in vitro* kinase assay to determine if PXR was a direct target of AMPK. In our *in vitro* study, we used purified active AMPK with purified PXR-GST as a substrate and found that the PXR was not phosphorylated by the AMPK (Fig. 5, lane 7). We did observe AMPK autophosphorylation (Fig. 5, lane 4), and this was out-competed by the SAMS peptide (an AMPK synthetic substrate) (Fig. 5, lanes 9–11), indicating that the kinase assay worked as expected. This suggests that although AMPK plays an essential role in regulating PXR and the kinase activity of AMPK is required, PXR is not a direct substrate of AMPK.

### AMPK activation potentiates drug sensitivity

**Figure 6:**
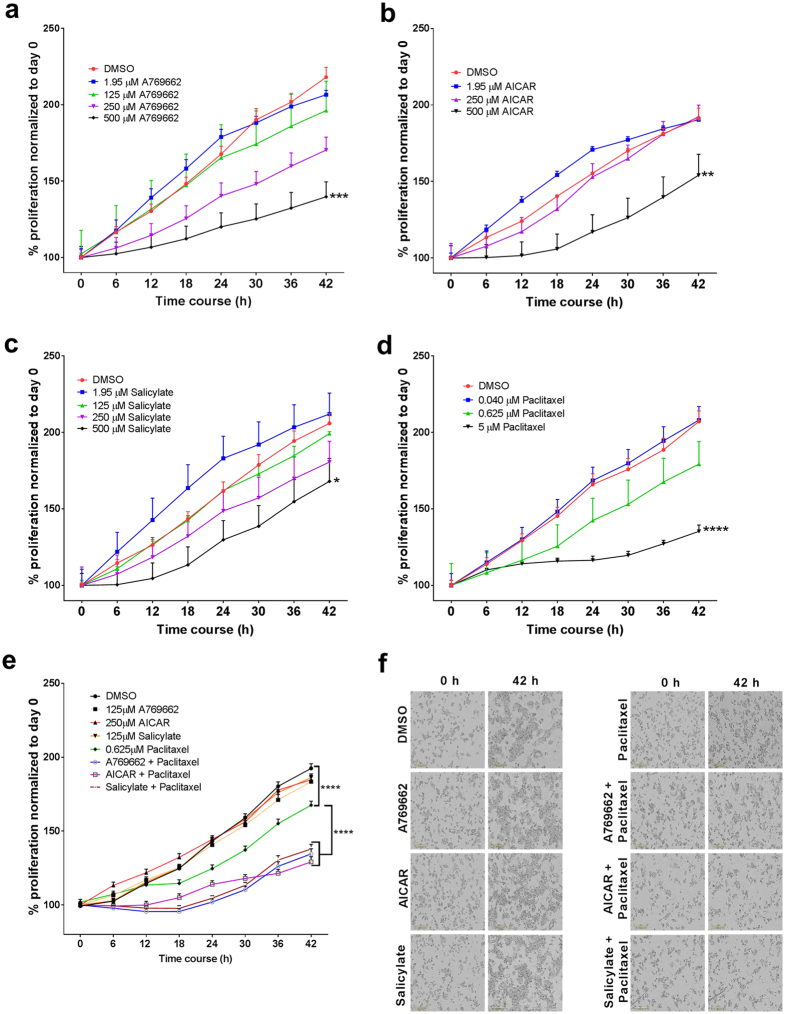
AMPK activation sensitizes cells to drug. (**a–c**) HepG2 cells were treated with increasing concentrations of AMPK activators, and cell proliferation was assessed for 42 h by using live-cell microscopy. (**d**) HepG2 cells were treated with increasing concentrations of paclitaxel, and cell proliferation was assessed for 42 h. (**e,f**) HepG2 cells were treated with concentrations of AMPK activators that had no significant effect on cell proliferation, together with 0.625 μM paclitaxel. Changes in cell numbers (in **e**) after 42 h are shown as percentages of the cell number at the starting time point. The images in (**f**) show the cell morphology at 0 and 42 h. The statistical analysis is a comparison of the endpoints of the treatment groups to that of the control group (DMSO treatment), unless otherwise indicated. The *P*-values were determined using ANOVA with Tukey’s HSD test: *P < 0.05, **P < 0.01, ***P < 0.001, ****P < 0.0001, n = 3. Results are expressed as the mean ± SE.

We used live-cell imaging to assess cell proliferation and sensitivity to paclitaxel. Cells were treated with increasing concentrations of AMPK activators, which slowed cell growth in a dose-dependent manner (Fig. 6a–c). This is consistent with the results of previous studies showing that high concentrations of AMPK activators induce cell death in different cell types^46^,^52^,^53^,^54^. Paclitaxel also slowed cell growth but did not completely inhibit cell proliferation at the concentrations used (Fig. 6d). To determine the effect of AMPK activation on the cell response to paclitaxel, we combined low concentrations of AMPK activators that did not independently affect cell proliferation with 0.625 μM paclitaxel that had only a minimal effect on cell proliferation. With these combination treatments, we observed a reduction of approximately 65% in cell proliferation, whereas treatment with paclitaxel alone resulted in a reduction of only approximately 25% (Fig. 6e). In Fig. 6f, live-cell images of the same fields acquired 42 h apart support the quantitative data regarding the cell proliferation. AMPK activation inhibited PXR activity (Fig. 4a–c). If the increased sensitivity of cells to the combination of AMPK activators with paclitaxel depends on the inhibition of PXR, elevated PXR levels may abolish such combination effects. Indeed, when PXR was ectopically overexpressed, cells became less sensitive to the combined effects of 125 μM A769662 and 0.625 μM paclitaxel, suggesting that the combination effects are at least partly PXR-dependent (see Supplementary Fig. S5).

Furthermore, to directly test if different glucose concentrations affect cellular sensitivity to drug, cells were incubated in 5 μM paclitaxel with either low (5.5 mM glucose), medium (25 mM glucose) or high (50 mM glucose) glucose concentrations, and cell proliferation was assessed. Cells in high glucose proliferated significantly more than cells in medium or low glucose condition, suggesting that high glucose levels decrease cellular sensitivity to paclitaxel (see Supplementary Fig. S6a). The effects of high glucose is PXR-dependent, as knocking down PXR using siRNA abolished the effect of high glucose and resensitized the cells to paclitaxel (see Supplementary Fig. S6b). Together, these data suggest that activating AMPK resulted in decreased PXR activity; hence, the cells were more sensitive to paclitaxel. The increased drug sensitivity caused by PXR inhibition was rescued by overexpressing PXR, which is consistent with the function of PXR in decreasing the sensitivity of cancer cells to chemotherapeutic drugs^55^,^56^,^57^,^58^. Additionally, high glucose desensitizes cells to paclitaxel in a PXR-dependent manner.

## Discussion

**Figure 7:**
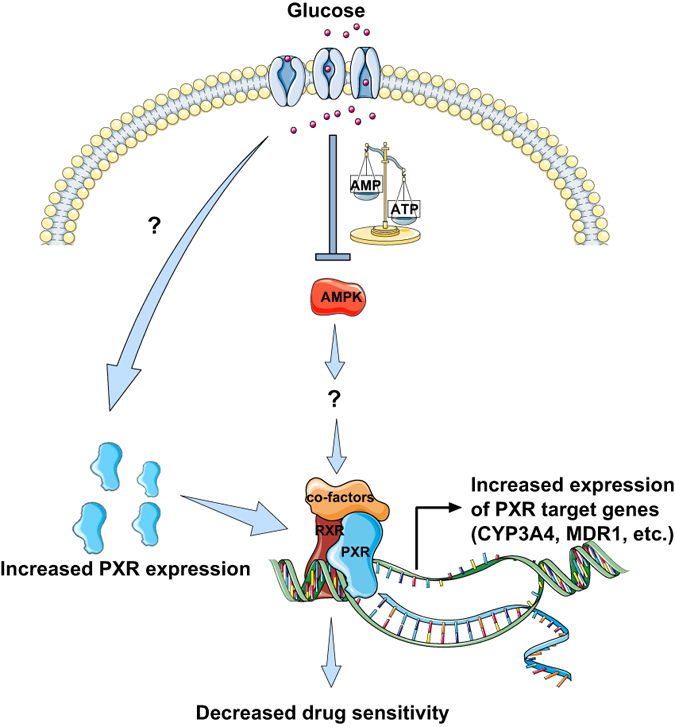
Proposed mechanism of glucose-dependent regulation of PXR activity. The schematic illustrates how glucose enhances both PXR transcription and PXR activity and increases the expression of PXR target genes. High glucose levels in the cell inactivate AMPK, and the inactivation of AMPK causes an upregulation of PXR activity. Increased PXR activity increases the transcription of PXR targets such as CYP3A4 and MDR1. The increased expression of drug transporters and DMEs potentially reduces the availability of drugs in the cell, resulting in decreased drug efficacy. The solid arrows represent activation, and the blunt arrow represents inhibition.

Our study demonstrated that glucose enhances PXR transcriptional activity by inhibiting AMPK activation. Additionally, we showed that PXR expression increases in response to high glucose in an AMPK-independent manner. Together, these two independent mechanisms maximally activate PXR and increase the expression of drug transporters and DMEs. The repression of glucose-induced PXR activity by AMPK activators suggests that AMPK is involved in the negative regulation of glucose-dependent induction of PXR. This is the first study to have shown a link between increased glucose levels, AMPK downregulation, and PXR upregulation. Our mechanistic investigation demonstrated that PXR is not a direct substrate of AMPK, suggesting the existence of an intermediary in the AMPK-PXR link. Our data indicate that the pharmacologic activation of AMPK made HepG2 cells more sensitive to paclitaxel, and as a result, their proliferation was greatly inhibited (Fig. 7).

The steady-state levels and induction of DMEs and drug transporters vary with diverse nutrients and different energetic and pathologic conditions^9^. Altered patterns of induction of CYP2B and CYP3A, which play a major role in the metabolism of over 50% of medications on the market, and the various drug transporters may result in differing drug pharmacokinetics. Therefore, understanding the molecular basis of the regulation of PXR, the driver of DMEs and drug transporters, under different pathophysiologic conditions is crucial for predicting drug pharmacokinetics and the response of individual patients to drugs.

In a recent study by Davidson *et al*. of long-term exposure of human hepatocytes to abnormal glucose levels, the expression of CYP1A2, CYP2B6, CYP2C19, and CYP3A4 was significantly increased after the cells were incubated for 10 or 18 days in hypoglycemic culture medium, as compared to a normoglycemic control^59^. The low-glucose conditions in that study apparently had effects similar to those we observed under high-glucose conditions in our own study. It is crucial to note that Davidson *et al*. used a micropatterned co-culture system of primary human hepatocytes and 3T3-J2 murine embryonic fibroblasts to maintain the insulin-sensitive glucose metabolism over the long term. They also used cells grown in different conditions over an extended period. In our current study, however, we consistently observed upregulation of PXR and CYP3A4 in high-glucose conditions and showed that the pharmacologic activation of AMPK robustly repressed glucose-induced PXR transcriptional activity. The discrepancy between our observations and those of Davidson *et al*.^59^ could be due to the differences in the experimental systems or cell types used (HepG2 cancer cells vs. primary hepatocytes).

Cancer cells are known to engage unique metabolic programs to satisfy their ATP requirements and biosynthetic needs. This shift in metabolism, known as the Warburg effect, helps cancer cells maintain their cell division rate^60^. Fang *et al*. previously reported an important mechanism by which AKT signaling increases ATP consumption and activates a rate-limiting enzyme in glycolysis that is otherwise inhibited by an elevated ATP:AMP ratio. Their study suggests there is a process by which proliferating cells integrate growth signals with their energy status to enable increased glucose uptake to support cell proliferation^61^. This may explain the differences we observed between the various cancer cell lines and normal primary hepatocytes in terms of their response to glucose in activating PXR. Differences in glucose uptake or use may play a role in these responses. In addition, cancer cells can potentially hijack this mechanism to increase drug metabolism in order to increase their survival. More importantly, AMPK activation and PXR inhibition induced by glucose deprivation were reversed by AMPK inhibitors in a concentration-dependent manner. The role of AMPK in maintaining the energy balance by inactivating ATP-consuming anabolic processes and activating ATP-generating catabolic processes under conditions of depleted cellular ATP supports our data regarding the inactivation of the drug metabolism pathway in low-glucose conditions. Drug metabolism is not an essential cell-fitness pathway when cells are in a low-energy state. Although active AMPK does not directly phosphorylate PXR, signaling molecules downstream from the AMPK pathway may function as the switch controlling the status of PXR activation. Masuyama and colleagues recently showed that the knockdown of PXR in ovarian cancer cells strongly inhibited the augmentation of MDR1 expression and PXR-mediated transcription by PXR ligands and significantly enhanced cell growth inhibition and apoptosis in the presence of paclitaxel or cisplatin^56^. An earlier report showed that siRNA targeting MDR1 could re-sensitize paclitaxel-resistant ovarian cancer cells^62^. These findings, together with our latest results, suggest that inhibiting PXR by activating AMPK either through pharmacological manipulation or by imposing energy stress, a pathway that regulates PXR activity, can augment the sensitivity of cells to anticancer agents or even overcome resistance to them. Although the currently available data point in this direction, the roles of xenobiotic receptors in drug resistance to anticancer agents require further study.

In conclusion, our results show that a high-glucose condition is a potent inducer of PXR transcription and PXR activity, at least in part through the inhibition of AMPK. We also observed that CAR is upregulated in response to high-glucose condition. Interestingly, the other nuclear receptors tested were not regulated in this manner suggesting that the regulation of drug metabolism by cellular energy status is in part mediated by PXR and probably also by CAR. The AMPK pathway appears to be important for regulating glucose-induced PXR activity, but it does not affect the glucose-dependent regulation of PXR expression. This suggests that PXR is regulated through a different mechanism at the transcriptional level, and we are currently investigating this aspect. Previous studies have shown that AMPK plays a role in phenobarbital-mediated CYP2B induction^63^. Even though AMPK regulates CAR signaling, the results from the various studies of this interaction have been contradictory. One group reported that AMPK caused CAR activation and induced CAR nuclear translocation, whereas another group reported the exact opposite^29^,^30^,^31^. This contradiction may be attributed to the known species-specific nature of CAR. How AMPK affects CAR activity, whether CAR affects drug sensitivity of cells in high-glucose milieu, and the molecular mechanism responsible for the role of AMPK in high glucose-induced, PXR-dependent decreased drug sensitivity, warrant further investigation. Although the AMPK pathway may not be the sole regulator of PXR as it relates to cellular energy status, our present observations, in conjunction with data obtained earlier^13^,^14^, contribute to an improved understanding of drug metabolism and drug clearance in diverse pathophysiologic and nutrient status conditions, including obesity, fasting, and metabolic diseases.

## Methods

### Materials

The C3A clone and parental HepG2 liver carcinoma cells, along with the LS180 colon cancer cells, were obtained from the American Type Culture Collection (ATCC, Manassas, VA). Primary human hepatocytes were obtained through the Liver Tissue Cell Distribution System (Pittsburgh, PA). Fetal bovine serum (FBS) was obtained from HyClone (Logan, UT). G418 and other cell-culture reagents were obtained from Invitrogen (Carlsbad, CA). Anti-mouse IRDye and anti-rabbit IRDye secondary antibodies were purchased from LI-COR Biosciences (Lincoln, NE). Anti-PXR antibody (clone H11), AMPK siRNA (sc-45312), and control siRNA (sc-37007) were purchased from Santa Cruz Biotechnology (Dallas, TX). PXR smart pool siRNA (SO-2505311G) and nontargeting siRNA were purchased from GE Dharmacon (Lafayette, CO). Anti-CYP3A4 antibody, clone K03, was previously described^64^. Anti-AMPK and anti-phosphorylated AMPK antibodies were purchased from Cell Signaling (Beverly, MA). Anti–β-actin (clone AC-15) and anti-MDR1 antibodies were obtained from Sigma-Aldrich (St. Louis, MO). Rifampicin, paclitaxel, T0901317, metformin, UCN-01, salicylate, and protease inhibitor cocktail and phosphatase inhibitor II were obtained from Sigma-Aldrich. AICAR, Cdk1/2 inhibitor II, and the indirubin derivative E804 were obtained from EMD Millipore (Billerica, MA). A769662 was obtained from ApexBio (Houston, TX). Dimethyl sulfoxide (DMSO) and glucose solution were purchased from Fisher Scientific (Pittsburgh, PA). Luciferase and Cell Tox Green reagent were purchased from Promega (Madison, WI). AMPK plasmids (WT, Addgene 79010; KD, Addgene 79011) were purchased from Addgene (Cambridge, MA), which were deposited by Dr. Reuben Shaw^65^. The PXR expression construct, FLAG-hPXR in which hPXR is tagged with the FLAG epitope (N-DYKDDDDK-C) at the N terminus has been described previously^66^. Purified GST was purchased from Protein One (Rockville, MD), and purified PXR-GST was purchased from LifeSpan Bioscience (Seattle, WA). The kinase inhibitor library was purchased from EMD Millipore. SAMS peptide (HMRSAMSGLHLVKRR-NH2) was purchased from Anaspec (Fremont, CA). ATP was purchased from Santa Cruz Biotechnology. Active AMPK (A1/B1/G2) was purchased from SignalChem (Richmond, BC, Canada). AICAR was also purchased from Selleck Chemicals (Houston, TX). ADP-Glo reagent was purchased from Promega. Tris-HCl, pH 7.5 (1 M), DTT (1 M), bovine serum albumin (BSA), Tb-anti-GST, and GST-PXR-LBD were purchased from Invitrogen. MgCl_2_ (1 M) was purchased from Fisher Scientific. The 384-well white solid plates were purchased from PerkinElmer (Waltham, MA) and the 384-well low-volume black assay plates were obtained from Corning Inc. (Tewksbury, MA). BODIPY FL vindoline was prepared in-house^49^.

### Cell culture

All cell lines were maintained in a humidified incubator at 37 °C with 5% CO_2_. A clone of HepG2 cells stably over-expressing FLAG-hPXR (in pCDNA3.1 vector) and CYP3A4-luciferase reporter (in pGL3 vector) (HepG2 PXR CYP-luc clone 1) (previously described as PXR clone 1 [ref. 66]) was generated and maintained in double-selection medium containing 500 μg/mL G418 and 200 μg/mL hygromycin (Life Technologies). Primary human hepatocytes were maintained in Williams E medium containing Primary Hepatocyte Maintenance Supplement (Life Technologies) and medium was changed to DMEM for experimentation. Cells collected for RNA and protein extraction were detached by scraping. For all luminescence-based assays, the cells were plated using phenol-red–free DMEM medium (Life Technologies). Cells were routinely verified to be mycoplasma-free by using the MycoProbe Mycoplasma Detection Kit (R&D Systems).

### Protein extraction and Western blot analysis

Protein was isolated from cells by incubating them in Pierce RIPA lysis buffer with added Halt Protease Inhibitor cocktail (Thermo Fisher Scientific) and Phosphatase Inhibitor Cocktail (Sigma) for 30 min on ice then sonicating the lysate for 10 s at 50% amplitude to shear the DNA. The protein concentration was measured using the Pierce BCA Protein Assay (Thermo Fisher Scientific) in accordance with the manufacturer’s instructions. Protein lysates were resolved on NuPAGE 4–12% SDS-PAGE gradient gels (Life Technologies). After electrophoresis was completed, the proteins were transferred from the gels to nitrocellulose membranes with an iBlot Dry Blotting System (Life Technologies). All membranes were blocked with Odyssey Blocking Buffer (LI-COR Biotechnology, Lincoln, NE). All antibodies were diluted in Odyssey Blocking Buffer. The secondary antibodies were diluted in TBS. All Western blot imaging was performed using a LI-COR Odyssey Infrared Imaging System.

### RNA extraction and quantitative real-time PCR

RNA was extracted using Maxwell simplyRNA Kits and a Maxwell 16 Instrument (Promega). RNA concentrations were measured using a NanoDrop 8000 UV-Vis Spectrophotometer (Thermo Fisher Scientific). All cDNA used in mRNA quantitative real-time PCR (qPCR) analyses was synthesized from extracted RNA by using the SuperScript VILO cDNA Synthesis Kit (Life Technologies) in accordance with the manufacturer’s protocol. The mRNA expression data were generated using Applied Biosystems TaqMan assays (20×) and Fast Advanced Master Mix (Life Technologies). Thermal cycling for qPCR was performed with an Applied Biosystems 7900HT Fast Real-Time PCR system (Life Technologies) in accordance with the TaqMan Fast protocol. Gene expression was normalized to the housekeeping gene 18 S ribosomal RNA (18 S), the expression of which did not vary in the different cell lines as a function of the glucose levels. Data are shown as the mRNA fold change (2^−ΔΔCT^) relative to the mRNA level of the corresponding transcript in the control samples as indicated. Each experiment was performed at least three times, and all samples were analyzed in triplicate.

### Luciferase assays

HepG2 PXR CYP-luc cells grown in flasks were trypsinized and plated in 96-well plates (CulturPlate-96, PerkinElmer) at 20,000 cells/well. Cells were treated as described above. PXR activity was determined as a measure of CYP3A4- luciferase reporter activity. The luciferase assay was performed in accordance with the manufacturer’s protocol. Briefly, a volume of the reagent equal to the volume of the medium on the cells was added and the plates were incubated at room temperature for 30 min with gentle agitation. The luminescence signal was measured with an EnVision plate reader (PerkinElmer), and the fold change in PXR activation was calculated.

### AMPK kinase assay

Recombinant active AMPK (400 ng) and purified PXR-GST (200 ng) were incubated in the presence of 300 μM nonradioactive ATP (Sigma-Aldrich) in AMPK kinase buffer (400 mM Tris, pH7.5, 20 mM MgCl_2_, 0.1 mg/mL BSA, 50 μM DTT). The reaction mixture was incubated at 30 °C for 1 h with gentle agitation, then the reaction was stopped by adding sample loading buffer and boiling for 5 min. The entire reaction volume was resolved on SDS-PAGE and immunoblotted with the indicated antibodies.

### AMPK inhibitor and activator screen

In the primary screening to identify potent AMPK inhibitors, a library of 240 putative kinase inhibitors (EMD Millipore) at concentrations of 2 μM, along with DMSO controls, was incubated with or without 10 ng AMPK protein in 15 μL reaction mixture (50 μM ATP and 50 μM SAMS peptide in the assay buffer of 40 mM Tris, pH 7.5, 20 mM MgCl_2_, 0.1 mg/mL BSA, 50 μM DTT) at room temperature for 60 min in 384-well white solid-bottom plates. An ADP-Glo assay was then performed. The luminescence signal for each well was measured with a PHERAstar *FS* plate reader (BMG Labtech, Durham, NC). The DMSO concentration was 0.2% for all assay wells. The signal values from DMSO without AMPK and with AMPK were used as positive (100% inhibition) and negative (0% inhibition) controls, respectively. The activity of each well was normalized to that of the positive and negative controls. Those 32 chemicals with %inhibition values of at least 80% were selected for further dose-response confirmation. In the dose-response characterization of potent AMPK inhibitors and activators, titrations of the 32 selected AMPK inhibitors from the primary screening and three selected putative AMPK activators (A-769662, salicylate, and AICAR), along with DMSO controls, were incubated with or without 10 ng AMPK protein in 15 μL reaction mixture (50 μM ATP and 50 μM SAMS peptide in the assay buffer of 40 mM Tris, pH 7.5, 20 mM MgCl_2_, 0.1 mg/mL BSA, 50 μM DTT) at room temperature for 60 min in 384-well white solid-bottom plates. An ADP-Glo assay was then performed. The luminescence signal for each well was measured with a PHERAstar *FS* plate reader. The DMSO concentration was 0.2% for all assay wells. The signal values for DMSO without AMPK and with AMPK were used as positive (100% inhibition) and negative (0% inhibition) controls, respectively. The activity of each well was normalized to that of the positive and negative controls. The results for each chemical were fitted into a one-site competitive binding equation with GraphPad Prism to derive the IC_50_ values.

### Cell proliferation assays

Real-time cell growth in response to the various treatments was measured as the degree of cell confluence in culture plates and was determined using an IncuCyte ZOOM live-cell imaging system (Essen BioScience, Ann Arbor, MI). In brief, 10,000 cells/well were seeded in 96-well plates 24 h before the addition of the compounds. The cell density was assessed every 6 h throughout the experiment. Treatment with vehicle (DMSO) alone was used as a negative control. Cell proliferation curves were plotted using confluence values at specified time points for each treatment.

### Time-resolved fluorescence resonance energy transfer (TR-FRET) PXR ligand binding assay

PXR TR-FRET binding assays were performed as previously described^49^ with only minor modifications. Briefly, in black 384-well low-volume assay plates, concentrations of chemicals, T0901317, DMSO, or 10 μM T0901317 were incubated with 100 nM BODIPY FL vindoline, 5 nM GST-hPXR-LBD, and 5 nM Tb-anti-GST for 30 min in 20 μL/well assay buffer (50 mM Tris, pH 7.5, 20 mM MgCl2, 0.1 mg/mL BSA, 0.05 mM DTT). The TR-FRET signals (10,000 × 520 nm/490 nm) were then measured with a PHERAstar FS plate reader (BMG Labtech, Durham, NC) using a 340-nm excitation filter, a 100-μs delay time, and a 200-μs integration time. The final DMSO concentration was 1.1% for all assay wells. Signal values from 10 μM T0901317 and DMSO were used as positive (100% inhibition) and negative (0% inhibition) controls, respectively. The activity of each well was normalized to that of the positive and negative controls. The normalized results for each chemical were fitted into a one-site competitive binding equation with GraphPad Prism to derive the IC_50_ values. In the test, the positive control T0901317 had an IC_50_ value of 95.9 ± 7.0 nM, which is consistent with the value of 101.6 nM previously recorded under similar assay conditions^49^.

### Plasmid and siRNA transient transfection

For plasmid transfection, HepG2 cells were grown to 80% confluency. The transfection reagent Lipofectamine 2000 (Invitrogen) and 3 μg AMPK plasmids were pre-incubated at room temperature for 5 min at a ratio of 3:1 in serum-free Opti-MEM medium (Gibco). The mixture was then added to the cells in culture medium. The culture flasks were incubated at 37 °C for 24 h, then the cells were trypsinized and replated in 96-well plates for further analysis. For siRNA transfection, cells were plated as described above. The transfection reagent RNAi max (Invitrogen) at a ratio of 3:1 with 100 nmol/L nontargeting (ctrl siRNA) or targeting siRNA (AMPK and PXR), was pre-incubated at room temperature for 5 min in Dharmacon ACCELL siRNA delivery medium (Thermo Scientific). The mixture was then added to the HepG2 cells in culture medium. The culture flasks were incubated at 37 °C for 24 h, then the cells were trypsinized and replated in 96-well plated for further analysis.

### Statistical analysis

Data from at least three independent replicated experiments were pooled and quantitatively analyzed by one-way analysis of variance (ANOVA) plus Tukey’s honest significant difference (HSD) test and by Student’s *t*-test using GraphPad Prism 7.0 software. *P*-values of less than 0.05 were considered to indicate significance. Results are expressed as the mean ± SE.

## Additional Information

**How to cite this article:** Oladimeji, P. O. *et al*. Glucose-dependent regulation of pregnane X receptor is modulated by AMP-activated protein kinase. *Sci. Rep.* **7**, 46751; doi: 10.1038/srep46751 (2017).

**Publisher's note:** Springer Nature remains neutral with regard to jurisdictional claims in published maps and institutional affiliations.

## Supplementary Material

Supplementary Information

## Figures and Tables

**Table 1:** IC_50_ values of hits from compound screening.

	Compound	IC_50_ (M)
**Inhibitors**	GSK-3 inhibitor X	1.1e-006
	Syk inhibitor	1.803e-007
	JAK3 inhibitor VI	1.787e-007
	GSK-3 inhibitor XIII	1.47e-006
	Gö 6976	2.004e-006
	**Cdk1/2 inhibitor III**	**3.571e-008**
	Gö 6983	1.333e-006
	K-252a	2.094e-008
	SB 218078	2.01e-007
	PKR inhibitor	1.867e-008
	Flt-3 inhibitor II	1.241e-007
	**Indirubin derivative E804**	**8.121e-008**
	Indirubin-3′-monoxime	5.909e-007
	Staurosporine	1.106e-006
	Met kinase inhibitor	1.256e-006
	JAK inhibitor	3.859e-007
	SU6656	1.852e-007
	SU9516	2.072e-007
	Bisindolylmaleimide I	9.281e-007
	GSK-3 inhibitor IX	4.13e-007
	SU11652	4.185e-005
	Keratinocyte differentiation inducer	8.975e-008
	Indirubin-3′-monoxime, 5-Iodo-	5.405e-007
	Arcyriaflavin A	5.375e-006
	Bisindolylmaleimide III	2.837e-007
	Chk2 inhibitor	1.088e-007
	PI 3-Ka inhibitor	4.134e-008
	Ro-31–8220	6.163e-007
	Wee1/Chk1 inhibitor	1.136e-007
	**UCN-01**	**3.285e-008**
	Wee1 inhibitor	2.98e-008
	Dorsomorphin	1.268e-007
**Activators**	**A-769662**	**2.152e-007**
	**Salicylate**	**5.305e-005**
	AICAR	1.897e-006
