# Supplementary material for: Glucose-dependent regulation of pregnane X receptor is modulated by AMP-activated protein kinase
Source: Sci Rep. 2017 Apr 24;7:46751. doi: 10.1038/srep46751 (PMC5402287; doi:10.1038/srep46751)
Supplement: Supplementary Information [file srep46751-s1.pdf]

Supplementary Information for

Glucose-dependent regulation of pregnane X receptor is modulated by AMP-activated protein kinase

Peter O. Oladimeji[1], Wenwei Lin[1], C. Trent Brewer[1,2] and Taosheng Chen[1,2*]

[1]Department of Chemical Biology and Therapeutics, St. Jude Children's Research Hospital, Memphis, Tennessee 38105, USA.

[2]Integrated Biomedical Sciences Program, University of Tennessee Health Science Center, Memphis, Tennessee, USA.

*To whom correspondence should be addressed: Taosheng Chen, Department of Chemical Biology and Therapeutics, MS 1000, St. Jude Children's Research Hospital, 262 Danny Thomas Place, Memphis, TN 38105, USA. Tel: (901) 595-5937; Fax: (901) 595-5715; E-mail: taosheng.chen@stjude.org

Supplementary Figure 1. Glucose-dependent activation of PXR is not cell type specific. (a) Parental HepG2 cells were serum and glucose deprived for 24 h. Increasing concentration of glucose was re-introduced for 24 h after which CYP3A4 mRNA levels was assessed. (b) Serum and glucose were washed out of LS180 cells and immediately replaced with DMEM containing 3.125 mM glucose for 24 h. Increasing concentration of glucose was added for 24 h after which CYP3A4 mRNA levels was assessed. (c) Primary human hepatocytes from a single donor, and (d) Caco2 cells were serum and glucose deprived for 24 h. Increasing concentration of glucose was re-introduced for 24 h after which CYP3A4 mRNA levels was assessed. In a, b and d, the values represent the means of three independent experiments, and the bars denote the standard error of mean (SE). c is a single representation of the experiments performed in hepatocytes and statistical significance is comparison with DMSO. The values represent the means of three technical replicates, and the bars denote the standard error of mean (SE). The p value was determined using the ANOVA with Tukey's HSD test and statistical significance expressed as follows: *, $p < 0.05$; **,$p < 0.01$ ***, $p < 0.001$; ****, $p < 0.0001$.

Supplementary Figure 2. The effect of high glucose on gene expression is specific for PXR and CAR.

HepG2 cells were serum and glucose deprived for 24 h. 50 mM glucose was re-introduced for 24 h after which the indicated mRNA levels were assessed. The values represent the means of at least three biological replicates, and the bars denote the standard error of mean (SE). The p value was determined using the Student's t test and the comparison is between –glu and + glu for each gene. The statistical significance is expressed as follows: **,$p < 0.01$; ****, $p < 0.0001$.

Supplementary Figure 3. Cell-based assessment of AMPK activators and inhibitors. HepG2 cells were treated with increasing concentration of AMPK activators (upper panels). In the lower panels, HepG2 cells were deprived for 24 h and then treated with increasing concentrations of the indicated inhibitors. Whole cell lysates were resolved on SDS-PAGE and probed with the pT172 AMPK and AMPK antibodies.

Supplementary Figure 4. AMPK activators are not cytotoxic within the concentration range effective for activating AMPK, and AMPK inhibitors are weak PXR ligands. (a-c). HepG2 PXR CYP-luc cells were treated with AMPK activators to mimic conditions used in the luciferase assays. Cells were deprived and treated with glucose and indicated compounds for 24 h. Cell viability were measured by using CellTox Green reagent. No significant change in cell viability was observed between treatments. Viability of cells treated with DMSO and 50 mM glucose (control) was set to 100% in all assays. (d). PXR TR-FRET PXR assay was performed to determine if the identified AMPK inhibitors are strong ligands for PXR. PXR binding affinity was compared to the binding of T0901317, a strong PXR ligand.

Supplementary Figure 5. The combined effect of AMPK activator and paclitaxel is PXR-dependent. HepG2 cells were transfected with either empty vector (Vector) or FLAG-hPXR (PXR). Twenty-four hours after transfections, the two groups of cells were treated with DMSO as control, 125 μM A769662, 0.625 μM paclitaxel or a combination of 125 μM A769662 and 0.625 μM paclitaxel and live cell imaging was performed to assess cell proliferation. The values represent the means of three biological replicates at 42 h after compound treatment, and the bars denote the standard error of mean (SE). The p value was determined using the ANOVA with

Tukey's HSD test and the indicated statistical significance is a comparison of same treatment between groups (i.e. Vector vs PXR). The statistical significance is expressed as follows: ****, $p < 0.0001$. Inset: Western blot showing the expression of PXR.

Supplementary Figure 6. High glucose decreases drug sensitivity in a PXR-dependent manner. (a) HepG2 cells were treated with increasing concentrations of glucose with 5 μM paclitaxel, and cell proliferation was assessed for 90 h. Changes in cell numbers after 90 h are shown as percentages of the cell number at the starting time point. The statistical analysis indicated is a comparison of the endpoints between 50 mM and 5.5 mM glucose groups. (b) PXR siRNA or non-targeting (NT) siRNA was transfected into HepG2 cells. The cells were treated with 50 mM glucose (glu) with or without 5 μM paclitaxel. Changes in cell numbers after 90 h are shown as percentages of the cell number at the starting time point. The statistical analysis indicated is a comparison of the endpoints. The P-values were determined using ANOVA with Tukey's HSD test: **** $P < 0.0001$, $n = 3$. Results are expressed as the mean ± SEM

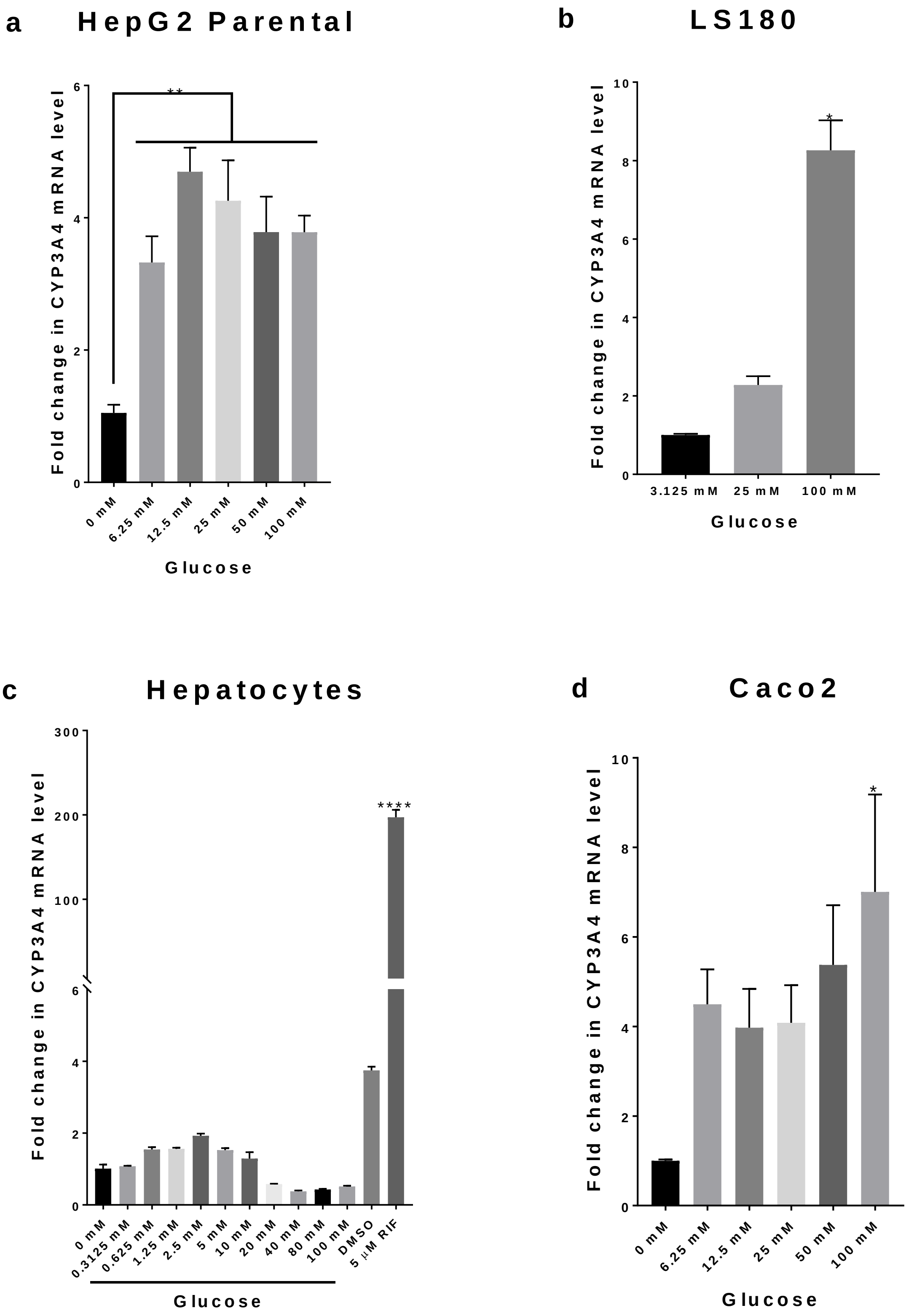
a
HepG2 Parental
Fold change in CYP3A4 mRNA level
0
2
4
6
**
0 mM
6.25 mM
12.5 mM
25 mM
50 mM
100 mM
Glucose
b
LS180
Fold change in CYP3A4 mRNA level
0
2
4
6
8
10
*
3.125 mM
25 mM
100 mM
Glucose
c
Hepatocytes
Fold change in CYP3A4 mRNA level
0
2
4
6
100
200
300
****
0 mM
0.3125 mM
0.625 mM
1.25 mM
2.5 mM
5 mM
10 mM
20 mM
40 mM
80 mM
100 mM
DMSO
5 μM RIF
Glucose
d
Caco2
Fold change in CYP3A4 mRNA level
0
2
4
6
8
10
*
0 mM
6.25 mM
12.5 mM
25 mM
50 mM
100 mM
Glucose

Fig. S1a-d

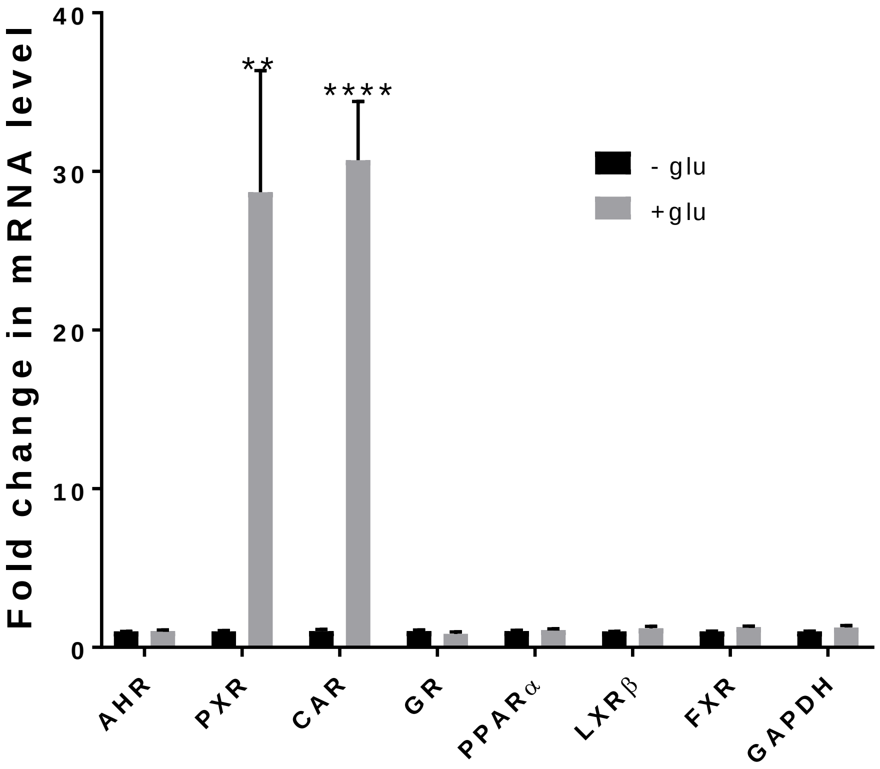

Fold change in mRNA level
40
30
20
10
0
**
****
- glu
+glu
AHR
PXR
CAR
GR
PPARα
LXRβ
FXR
GAPDH

Fig. S2

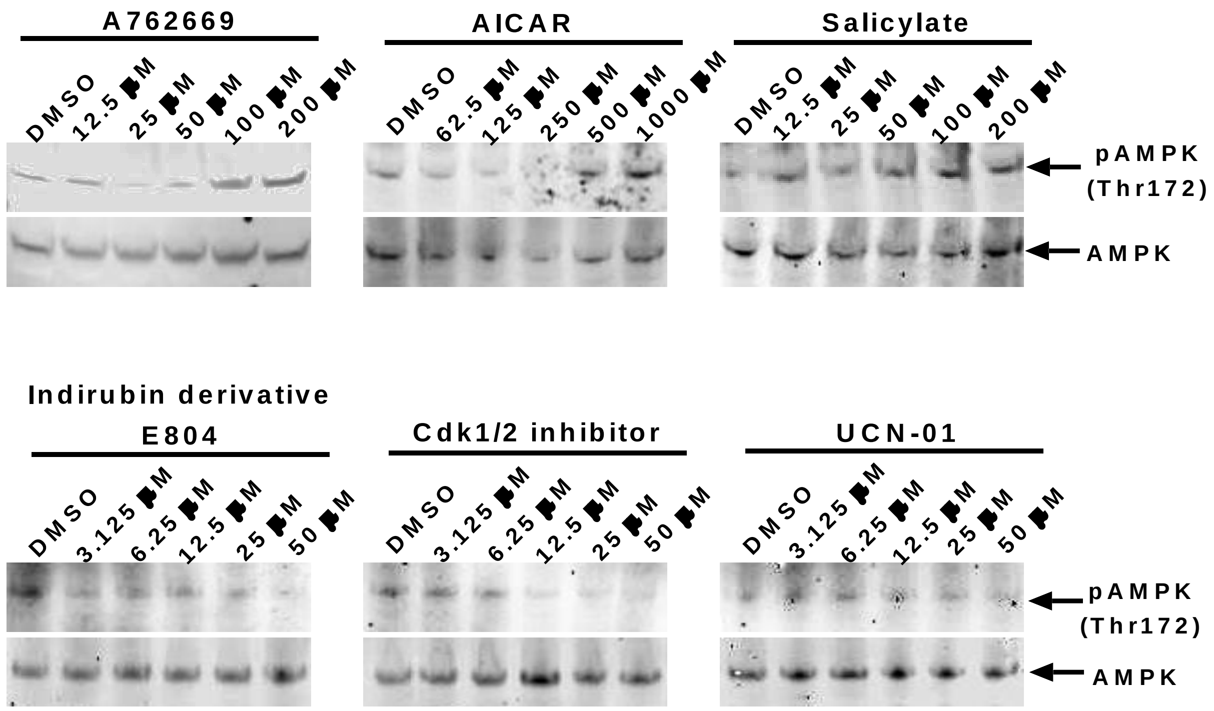
A762669
DMSO 12.5 μM 25 μM 50 μM 100 μM 200 μM
AICAR
DMSO 62.5 μM 125 μM 250 μM 500 μM 1000 μM
Salicylate
DMSO 12.5 μM 25 μM 50 μM 100 μM 200 μM
pAMPK (Thr172)
AMPK
Indirubin derivative E804
DMSO 3.125 μM 6.25 μM 12.5 μM 25 μM 50 μM
Cdk1/2 inhibitor
DMSO 3.125 μM 6.25 μM 12.5 μM 25 μM 50 μM
UCN-01
DMSO 3.125 μM 6.25 μM 12.5 μM 25 μM 50 μM
pAMPK (Thr172)
AMPK

Fig. S3

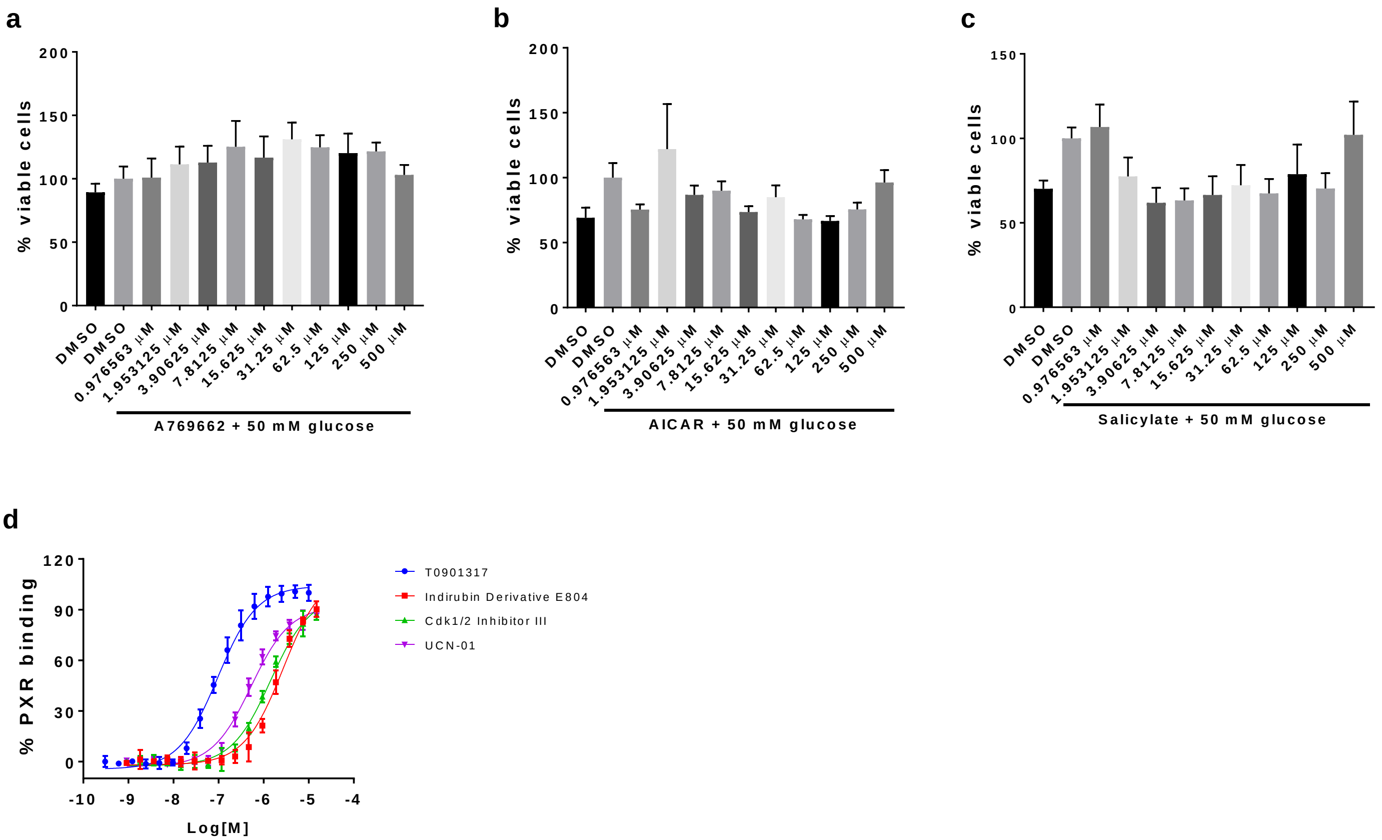
a
b
c
d
% viable cells
DMSO
0.976563 µM
1.953125 µM
3.90625 µM
7.8125 µM
15.625 µM
31.25 µM
62.5 µM
125 µM
250 µM
500 µM
A769662 + 50 mM glucose
AICAR + 50 mM glucose
Salicylate + 50 mM glucose
% PXR binding
Log[M]
T0901317
Indirubin Derivative E804
Cdk1/2 Inhibitor III
UCN-01

Fig. S4a-d

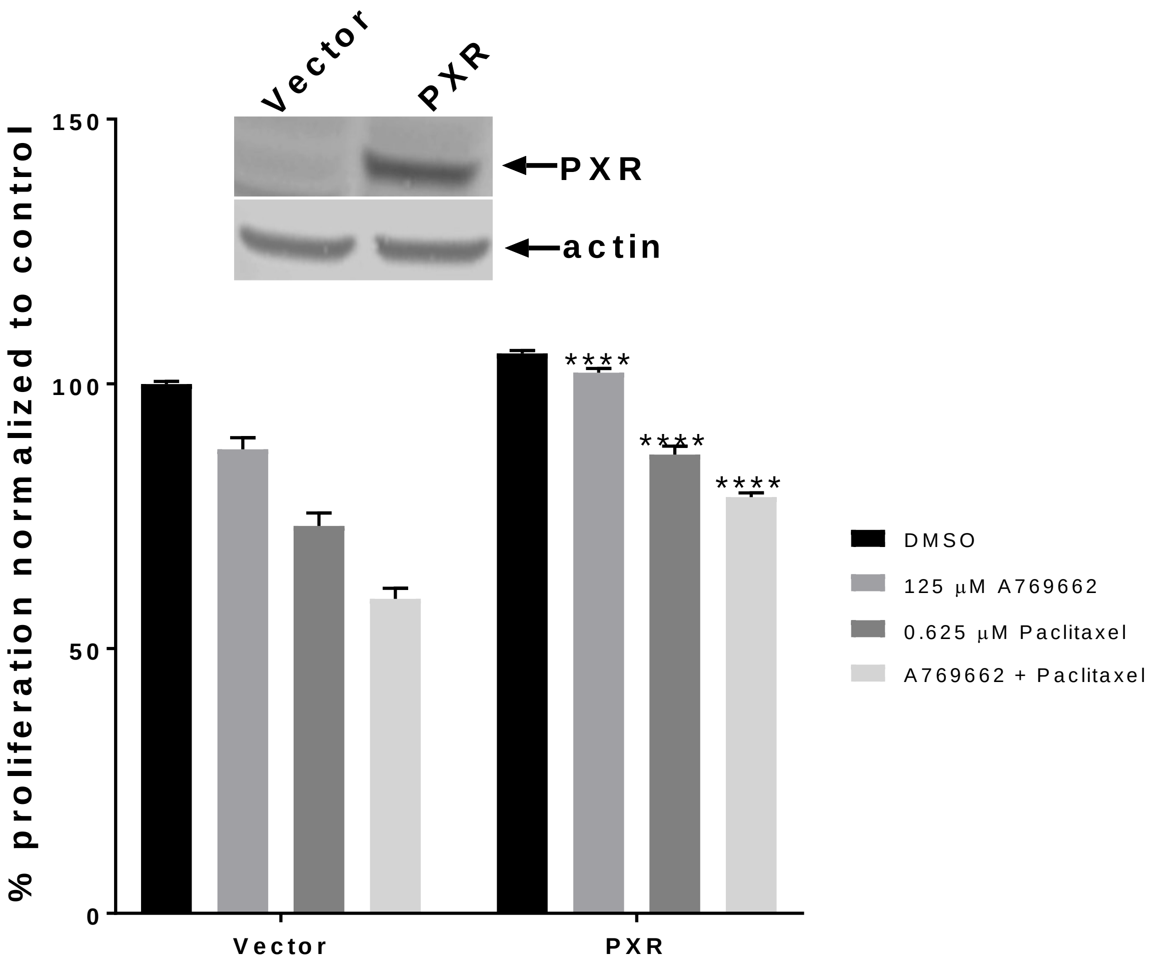
Vector
PXR
PXR
actin
% proliferation normalized to control
150
100
50
0
****
****
****
Vector
PXR
DMSO
125 µM A769662
0.625 µM Paclitaxel
A769662 + Paclitaxel

Fig. S5

a

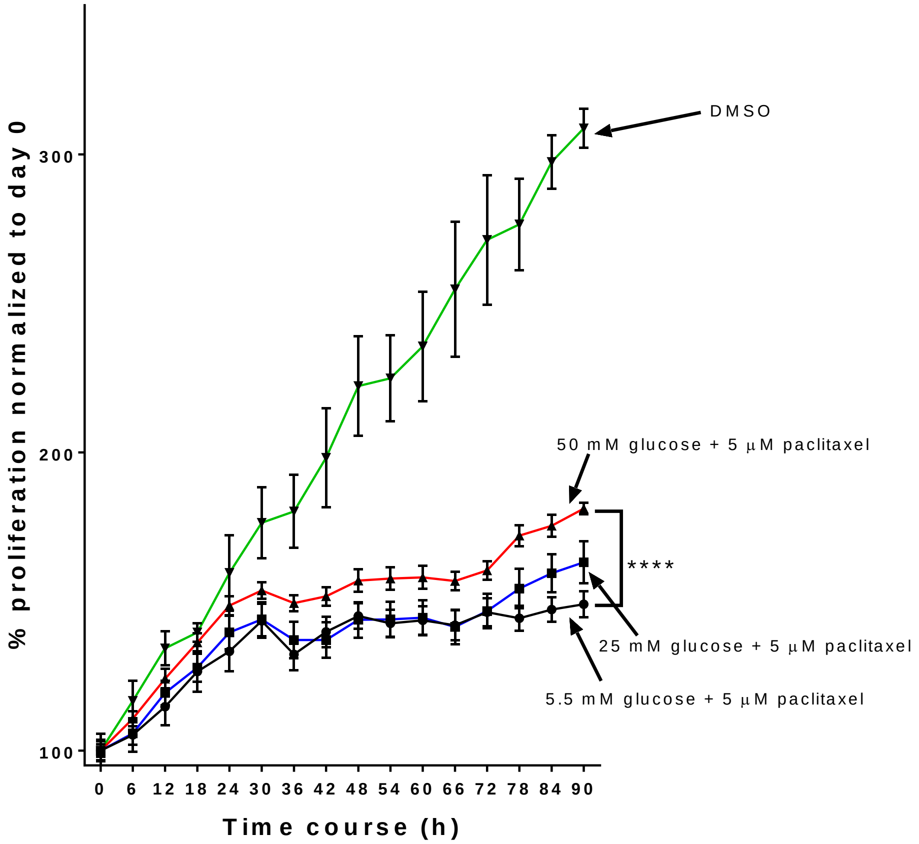
DMSO
50 mM glucose + 5 µM paclitaxel
25 mM glucose + 5 µM paclitaxel
5.5 mM glucose + 5 µM paclitaxel
****
% proliferation normalized to day 0
100
200
300
0 6 12 18 24 30 36 42 48 54 60 66 72 78 84 90
Time course (h)

b

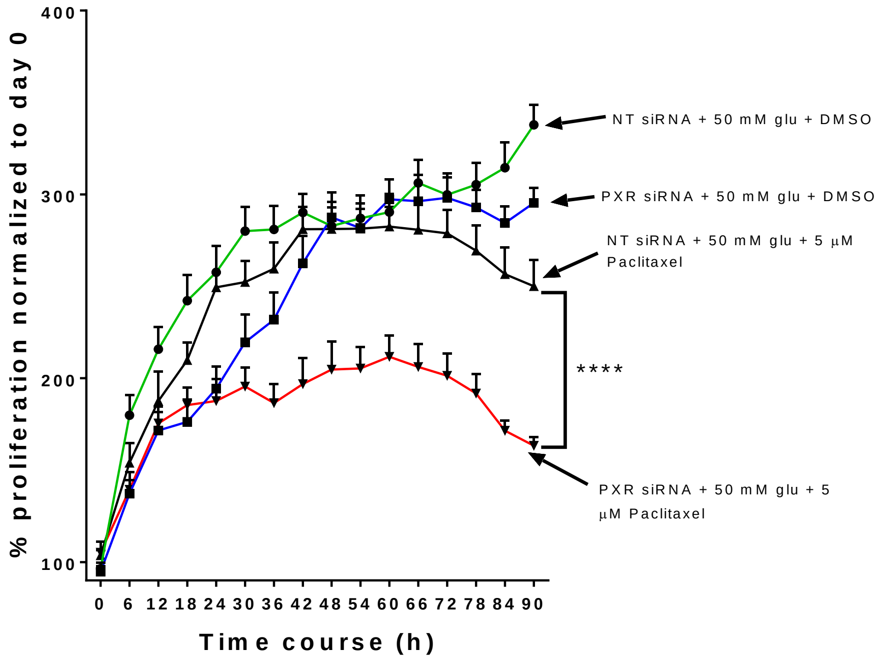
NT siRNA + 50 mM glu + DMSO
PXR siRNA + 50 mM glu + DMSO
NT siRNA + 50 mM glu + 5 µM Paclitaxel
PXR siRNA + 50 mM glu + 5 µM Paclitaxel
****
% proliferation normalized to day 0
100
200
300
400
0 6 12 18 24 30 36 42 48 54 60 66 72 78 84 90
Time course (h)

Fig. S6a-b
